# Liposuction Complications in the Outpatient Setting: A National Analysis of 246,119 Cases in Accredited Ambulatory Surgery Facilities

**DOI:** 10.1093/asjof/ojad107

**Published:** 2023-11-28

**Authors:** Lauren Valentine, Angelica Hernandez Alvarez, Allan A Weidman, Jose Foppiani, Natalie E Hassell, Nicholas Elmer, Paul Hwang, Sumedh Kaul, William Rosenblatt, Samuel J Lin

## Abstract

**Background:**

Suction lipectomy (liposuction) is a popular cosmetic surgical procedure performed in the United States, but little has been documented regarding perioperative complications due to its outpatient nature.

**Objectives:**

This cross-sectional study aims to analyze the most common complications that accompany liposuction-related procedures and importantly estimate the total complication rate occurring at ambulatory surgical facilities.

**Methods:**

Adult patients who experienced liposuction-related complications from 2019 to 2021 were identified in the reporting database of the global surgery accreditation authority, the American Association for Accreditation of Ambulatory Surgery Facilities (QUAD A). Patients were then divided by complication type and procedure location. Demographics and facility-specific variables were analyzed. Descriptive statistics were performed.

**Results:**

Overall, 984 patients were included, with a mean age of 44 years (interquartile range [IQR] 37-53) and a median BMI of 28.7 kg/m^2^ (IQR 25.7-32.2). The overall confirmed complication rate was found to be 0.40% (984/246,119). Unplanned emergency department presentation was the most common complication overall (24%). Wound disruption was associated with the longest median procedure length (261 min), and venous thromboembolism was associated with the highest median BMI (30.1 kg/m^2^). The Southeast had the most complications (431), which accounted for 13/21 deaths (61.9%). Out of all complications, death was associated with the highest average annual case volume (241).

**Conclusions:**

Procedures that involve liposuction are associated with a variety of medical and surgical complications. Given the high frequency and variability in how liposuction is performed, a thorough assessment of complications is critical to improve the safety of this procedure.

**Level of Evidence: 3:**

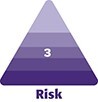

Suction lipectomy (liposuction) is one of the most commonly performed aesthetic surgery procedures, with over 491,000 operations performed in the United States in 2021.^1^ This procedure reduces the amount of subcutaneous adipose tissue in localized regions of the body, including but not limited to the face, arms, breasts, abdomen, waist, hips, buttocks, and thighs.^2,3^ The setting and method by which liposuction is carried out are highly dependent on both provider- and patient-specific factors. This procedure is offered by practitioners from various specialties in both the inpatient and outpatient settings. There are various wetting solutions that can be infiltrated, variable amounts of volume aspirated, and numerous cannulas that can be used—none of which are universally standardized.^3,4^ Additionally, liposuction is increasingly being added on as an adjunct to other cosmetic procedures, which may significantly increase the length of the overall procedure.^5^ Offering this procedure in-office or at ambulatory surgical facilities can increase patient access, comfort, and surgeon convenience as well as minimize cost, which has allowed liposuction to be performed as an outpatient procedure at increasing rates.^5^

Given the technical variability and predominately outpatient nature, the incidence of complications following cosmetic liposuction is challenging to assess based on the current literature with reported rates ranging from 0% to 10%.^6^ Local complications include contour irregularities, seroma, hematoma, and skin conditions such as wound dehiscence and necrosis.^7,8^ Systemic complications from liposuction can be potentially lethal and include complications such as pulmonary embolism (PE), fat embolism, sepsis, necrotizing fasciitis, and perforation of intra-abdominal organs.^8^ The reported incidence of death after liposuction varies widely, from 2.6 to 20.6 per 100,000.^9^ Given its popularity and lack of technical standardization, an analysis and understanding of the complications associated with this procedure are critical to improve the safety of this popular procedure.

Because most plastic surgeries today take place in ambulatory surgery centers or accredited office surgery facilities, there is a paucity of data regarding complications from liposuction in the ambulatory setting.^10^ The goals of this study were to attempt to find the highest accuracy of a true incidence of complications in the outpatient space and to analyze complications from ambulatory liposuction as reported in the Patient Safety Data Reporting (PSDR) from the American Association for Accreditation of Ambulatory Surgery Facilities (AAAASF), or QUAD A, a large global accreditation organization for outpatient surgery facilities. Understanding the complication profile and risk factors that accompany patients undergoing outpatient liposuction will enable evidence-based improvements in patient safety and outcomes in ambulatory plastic surgery.

## METHODS

### Study Design and Data Collection

This study was deemed exempt from full review by the institutional review board at our institution (2022D000071). QUAD A is an international accreditation organization that works with independent healthcare facilities to standardize the quality of care they provide. QUAD A currently accredits 2969 facilities overall, 937 of which are specifically plastic surgery facilities or multispecialty facilities that have self-declared as being primarily plastic surgery. Each quarter, facilities are required to submit every unplanned complication to the QUAD A PSDR system through an online module. This reporting of complications is mandatory for each participating surgeon at every facility. The surgeons participating are all board-certified in their respective specialties, but submissions are not isolated by specialty and thus may have been from nonplastic surgery practitioners. In each instance, the facility and/or surgeon are required to report information related to the patient (age, gender, height, weight, etc) and the surgical case (Current Procedural Terminology [CPT], length of case, etc). Inclusion of a note describing the complication is encouraged, but not required. The sequelae database generated from this reporting system, representing all complications reported at QUAD A–accredited facilities from 2019 to 2021, was utilized in this study. The patient data were deidentified and thus no consent was necessary. Additionally, an estimate of the total number of liposuction procedures performed at these facilities was provided by QUAD A team members utilizing facility data collected from the PSDR.

### Categorizing Complications

Within the QUAD A complication data, cases involving liposuction from January 2019 to December 2021 were identified by CPT codes 15876, 15877, 15878, and 15879. Each liposuction case entry was individually analyzed for patient demographics such as age and BMI, as well as procedure information including location of the body, length of operative time, and additional details written in the notes about each complication. Complications were then divided into the following categories: seroma, hematoma, wound disruption or dehiscence, wound infection, intraoperative complication, venous thromboembolism (VTE), unplanned hospital presentation to the emergency department (ED), unplanned reoperation, other complications, and death. The “other complication” category included alternative sequelae such as postoperative nausea, vomiting, and severe pain. Cases entered with unrealistic characteristics (ie, height >7 feet, age >110 years) or that were located outside of the United States were excluded from analysis. There were 179 cases in which the sequelae listed were unrelated to patient care (ie, operating room malfunctions that never reached the patient), which were also excluded. Lastly, there were 577 cases in which the complication note was completely missing. We were unable to categorize those complications, and they were thus excluded from the complication percentages. Percentages sum to greater than 100% due to some patients experiencing multiple complications.

### Statistical Analysis

Baseline characteristics were summarized using medians and interquartile ranges (IQRs) or frequency counts and percentages. We examined the frequency counts and rates of complications individually and stratified them using bar plots. We also assessed median length of procedure, BMI, and annual average liposuction volume stratified by complication categories using line plots. Statistical analyses were performed using R version 4.2.2 (R Foundation for Statistical Computing, Vienna, Austria).

## RESULTS

### Demographic Information and Baseline Characteristics

**Figure 1.**
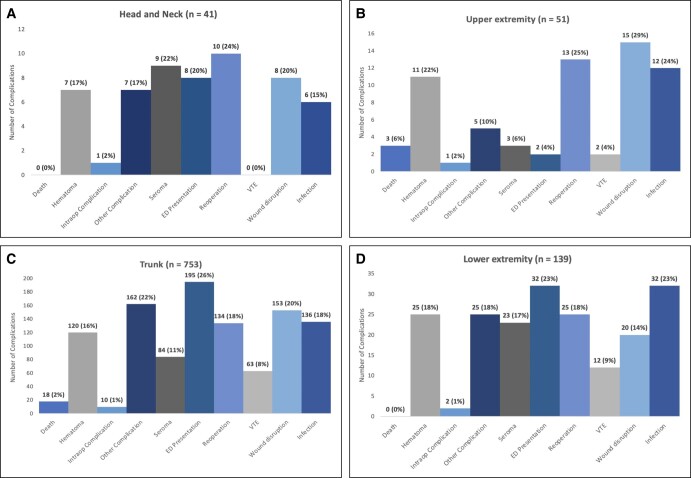
(A-D) Complications by site.

The total number of patients who underwent liposuction at QUAD A–accredited facilities over the 3-year period was 246,119. A total of 984 experienced detailed liposuction complications. The median age of the cohort was 44 years (IQR 37-53). The vast majority of patients had liposuction of the trunk (753, 77%), whereas 139 patients (14%) had lower extremity liposuction, 51 (5%) had upper extremity liposuction, and 41 (4%) had head or neck liposuction (Table, Figure 1).

**Table.** Baseline Characteristics of Patients and Complications

Characteristic	*n* = 984^a^
Age, years	44 (37-53)
BMI	28.7 (25.7-32.2)
Region	
Northeast	90 (9)
Midwest	106 (11)
Southwest	125 (13)
Southeast	431 (44)
West	232 (24)
*Site*	
Head and neck	41 (4)
Trunk	753 (77)
Upper extremity	51 (5)
Lower extremity	139 (14)
Length of procedure (min)	225 (150-305)
Annual average liposuction volume	165 (84-341)

^a^Median (IQR); *n* (%).

### Complications

**Figure 2.**
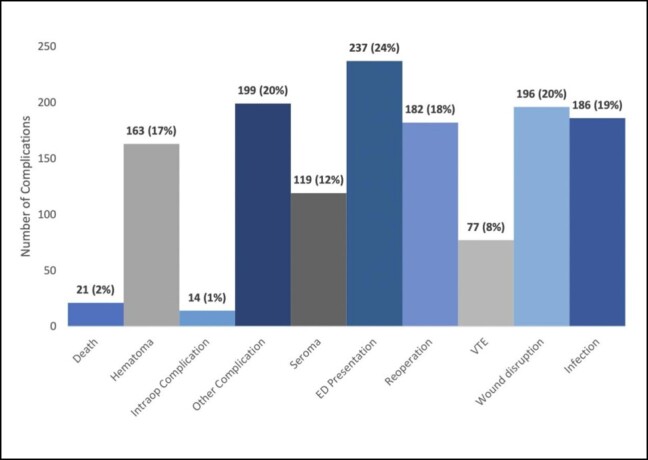
Total complications.

The medial age of our cohort was 44 years (range, 19-83 years; IQR, 37-53 years). The overall complication rate from documented and confirmed patient-related complications was 0.40% (984/246,119). When including 577 reported complications that had notes without any details, the overall complication rate increases to 0.63% (1561/246,119). Out of the 984 patients, a total of 1394 complications were documented. For example, if a patient experienced a seroma that required presentation to the hospital, the patient was recorded to have complications in both categories. The most common complication overall, experienced by 237 patients (24%), was an unplanned hospital presentation. This finding was followed by other complications (199, 20%), wound disruption (196, 20%), and wound infection (186, 19%). Only 14 patients (1%) had an intraoperative complication, whereas 21 patients died (2%). The overall mortality rate was 0.009% (21/246,119). The remainder of the complications are detailed in Figure 2.

**Figure 3.**
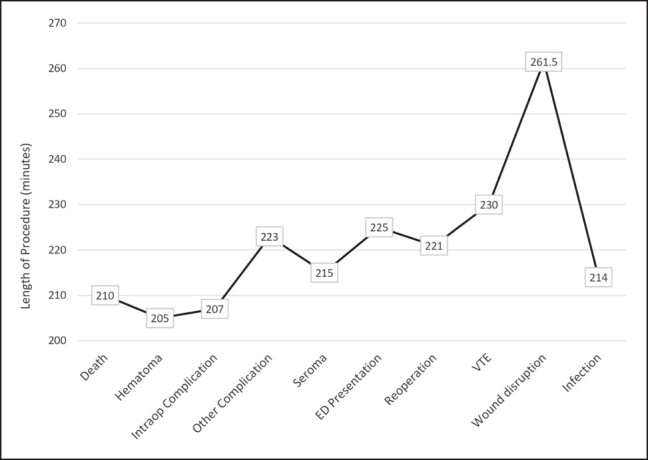
Median length of procedure by complications.

**Figure 4.**
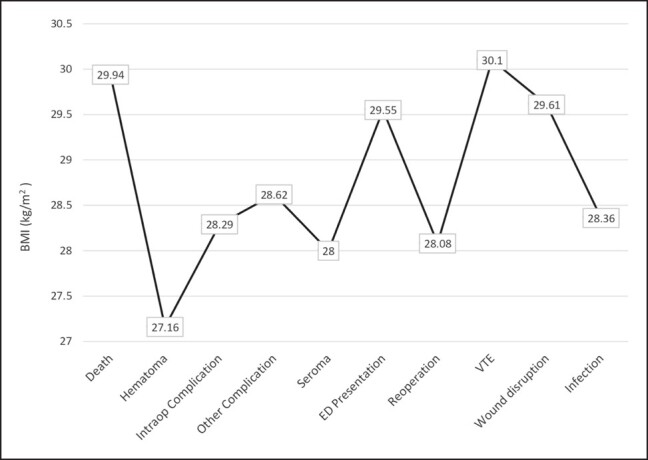
Median BMI by complications.

When assessing the complications by length of procedure, the complication associated with the longest procedure time was wound disruption, which had a median time of 261.5 min. This finding was followed by venous thromboembolism with a median time of 230 min, and unplanned hospital presentation with 225 min. The remainder of the complications by median length of operative time is shown in Figure 3. When assessing the most common complications by patient BMI, venous thromboembolism occurred in patients with the highest median BMI (30.1 kg/m^2^), followed by death in patients with a median BMI of 29.94 kg/m^2^. The remainder of the complications by BMI are shown in Figure 4.

**Figure 5.**
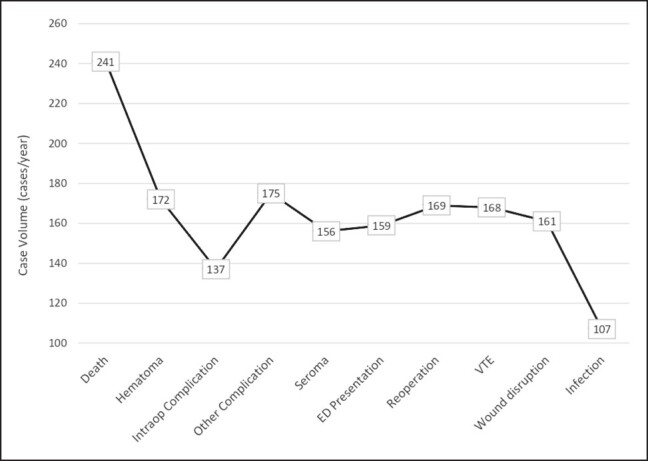
Complications by annual average liposuction volume.

Each complication was then analyzed with respect to case volume, with the average number of liposuction cases per year being calculated for each facility where the complication occurred. Strikingly, death was associated with the highest case volume, with facilities that experienced death as a complication performing an average of 241 liposuction cases per year. Following this were “other” complications occurring at facilities that perform an average of 175 cases per year, and hematoma occurring at facilities that perform an average of 172 cases per year. Infection was associated with the lowest case volume, with an average of 107 cases per year among facilities (Figure 5).

### Regional Breakdown

**Figure 6.**
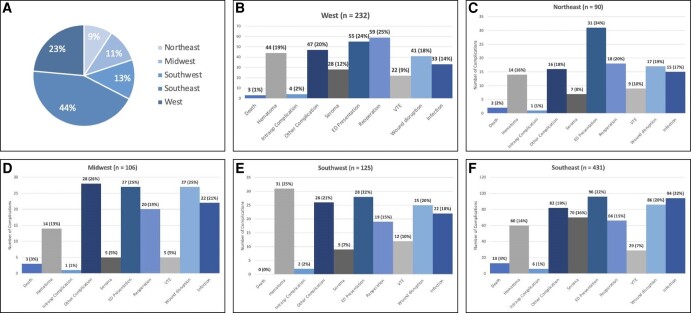
(A-F) Complications by region.

**Figure 7.**
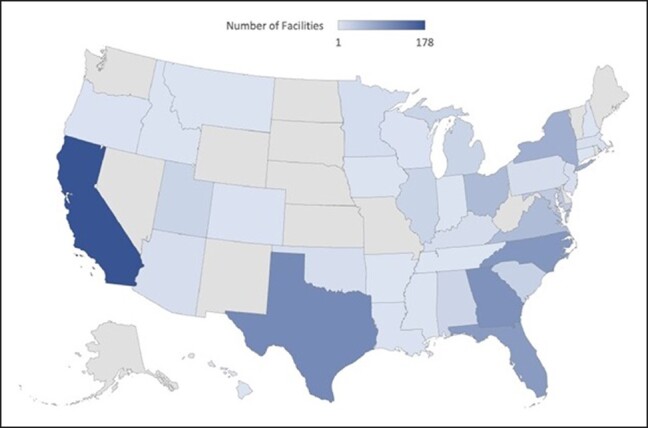
Density of facilities with complications.

Among all the complications, the Southeast had the most (431, 44%), followed by the West (232, 23%), Southwest (125, 13%), Midwest (106, 11%), and Northeast (90, 9%). In the Northeast and Southeast, unplanned hospital presentation was the most common complication (31 patients, 34% and 96 patients, 22%, respectively). Patients in the Midwest most commonly experienced “other” complications (28 patients, 26%), the Southwest most commonly experienced hematomas (31 patients, 25%), and the West most commonly had unplanned reoperations (59 patients, 25%). Most deaths occurred in the Southeast (13, 3%), which was collectively more than all the other regions combined (8 in total elsewhere). The remainder of the complications by region are shown in Figures 6 and 7.

## DISCUSSION

Aesthetic surgeries have transitioned from occurring primarily in hospital settings to being almost exclusively outpatient, with approximately 82% of procedures taking place in the ambulatory setting in 2020.^11^ Oppositely, the majority of research surrounding cosmetic surgery has been based on inpatient data. Therefore, this analysis of liposuction complications from procedures performed in outpatient QUAD A–certified facilities aims to open a window into the ambulatory surgery space on a national level. Notably, a study of liposuction complications in Germany found that an insufficient standard of hygiene was a major risk factor for severe complications.^9^ Given the strict hygiene standards in AAAASF facilities, an analysis of liposuction complications using the PSDR will enable an assessment of complications and risk factors while effectively controlling for hygiene. Additionally, surgeons operating in ambulatory surgery centers may be uniquely suited to enact quality improvement projects since they are less likely to be limited by hospital or institutional protocols.^12^ Thus, an understanding of complications and risk factors specific to the outpatient setting has the potential to inspire expeditious change.

The overall complication rate in our study ranged from 0.40 (984/246,119) to 0.63% (1561/246,119). This finding aligns with previously documented liposuction complication rates, which range from 0.26% to 0.7%.^6,13,14^ Similarly, the overall mortality rate was 0.009%, which is concordant with previous documentation of 0.002% to 0.02%.^13,15^ Our data revealed that the most common complication following ambulatory liposuction procedures nationwide was presentation to the hospital, seen in 24% of all patients who experienced complications. This finding is not unexpected, as prior studies have demonstrated that almost 4% of plastic surgery patients who undergo operations at ambulatory surgery facilities will present to the emergency department within 30 days.^16^ Additionally, many of the medical and surgical complications analyzed in this study also involved hospital presentation, such as venous thromboembolism, unplanned reoperation, and death. Given the initiatives over the past years to transition hospital-based care to ambulatory facilities, unanticipated hospital presentations following these procedures are used to weigh the safety and cost-efficacy of outpatient surgery.^17^ Our findings highlight that although most complications that arise at ambulatory surgery facilities can be managed in the outpatient setting, about one-quarter of patients still presented to the hospital emergency room for acute management.

One goal of this study was to assess patient-specific factors that were associated with certain complications. Our analysis revealed that the complications associated with the highest median BMI were venous thromboembolism (30.1 kg/m^2^) and death (29.94 kg/m^2^). Increasing BMI has previously been a well-established risk factor for clotting as well as for complications following liposuction.^6,18-20^ Venous thromboembolism was seen in 8% of patient complications and had an overall rate of 0.03% (77/246,119), which included deep vein thromboses (DVTs) and PE. This finding aligns with prior literature that found a DVT rate of 0.033% and a PE rate of 0.027%.^13^ Knowing that patients with higher median BMIs were the ones experiencing these thrombotic complications should encourage providers to utilize validated tools such as the Caprini Score to stratify patients preoperatively. Indeed, in a prior publication with QUAD A data, we found that there appeared to be an underutilization of preoperative DVT assessment and prophylaxis which led to a policy change across the facilities to assess and prevent venous thromboembolism events.^21^

Another important factor to consider when assessing complications was to ascertain if liposuction was performed independently or in conjunction with other cosmetic procedures. Due to the nature of the PSDR, we were unable to discern these details as all cases with liposuction CPT codes were included in our analysis. A systematic review by Kanapathy et al revealed that about one-third (863/2583) of patients receiving liposuction did so in conjuncture with other aesthetic procedures, indicating how common these combination procedures are.^22^ Previous literature has demonstrated that performing liposuction in combination with other procedures, while more efficient, is a risk factor for mortality.^6^ Similarly, prolonged surgical time was found to be associated with more postoperative complications (odds ratio 2.45, *P* < .001).^23,24^ In our analysis, the complication associated with the longest median length of procedure was wound disruption (261.5 min). The cases that have a longer operative time are likely not exclusively liposuction, but rather involve multiple adjunct procedures such as breast augmentations, abdominoplasties, or other invasive surgeries. Therefore, these combination procedures often involve multiple or longer incisions, thus increasing the risk of dehiscence and other disruptions of surgical wounds.

When assessing complications based on region, our analysis found that more complications occurred in the Southeast compared to anywhere else in the country (431 out of 984 total patients, 43.8%). Additionally, 13 out of 21 deaths that took place nationwide occurred in the Southeast. While it could be postulated that more cases of liposuction occur in the Southeast and thus account for the higher number, the American Society of Plastic Surgeons 2020 report states that only 19% of national liposuction cases occur in this region.^1^ The Southeast and South Florida in particular have been under scrutiny for concerns over patient safety regulations and high incidences of adverse events. This occurrence led to a legislative mandate in 2000 that all physicians report adverse events to a central collecting agency. In the 7 years following implementation, liposuction was found to be the single most common cause of death, accounting for 44.4% of cosmetic surgery mortalities.^25^ Similarly, a study by Pazmiño and Garcia highlighted how South Florida had an excessively high number of deaths associated following Brazilian butt lift procedures. When exploring why these procedures were so dangerous, they elucidated that the short surgical time was likely one of the most important contributors to mortality.^26^ An important additional finding in our study was that out of all the complications analyzed, death was associated with the highest average case volume. Facilities that experienced death as a complication performed an average of 241 liposuction cases per year, which was much higher than the average case volumes of the remaining complications (107-175 cases per year). Therefore, high case volumes and rapid patient turnover at a select few ambulatory surgery centers may be amounting to the disproportionately high complication and mortality rates in this region.

### Limitations

Given that some of the reporting fields were not mandatory in the PSDR, the QUAD A team used estimates based on historical data and national averages to arrive at a value for total case volume. Similarly, the percentages of complications were calculated including only patients who had clear and detailed reporting of their sequela. Therefore, the 577 reported complications that did not have any corresponding notes were excluded from the overall analysis. Additionally, the notes provided by each facility for each complication only provide information from a snapshot in time, thus lacking context and follow-up. For example, if a patient experienced a venous thromboembolism which was reported by the facility but then subsequently died, it would not be reported and thus would falsely depress the true complication rate. These limitations highlight the need for more thorough, detailed, and longitudinal tracking of complications to ensure utmost patient safety.

## CONCLUSION

This manuscript provided an overview of the complication profile that occurred following liposuction at QUAD A–facilitated ambulatory surgery facilities from 2019 to 2021. The overall complication rate was found to be between 0.40% and 0.63%, with the most common complication being presentation to the hospital. The complication associated with the highest median BMI was venous thromboembolism (30.1 kg/m^2^), the complication associated with the longest median length of procedure was wound disruption (261.5 min), and the complication associated with the highest average annual case volume was death. More complications, including deaths, occurred in the Southeast region than anywhere else in the country. With liposuction being one of the most common cosmetic surgery procedures performed in the United States, improved knowledge of the specific complication profile and risk factors among patients undergoing outpatient liposuction is key to enhancing patient safety and improving the quality of the ambulatory surgical experience.
